# Organizational readiness to implement task-strengthening strategy for hypertension management among people living with HIV in Nigeria

**DOI:** 10.1186/s43058-023-00425-3

**Published:** 2023-05-04

**Authors:** Juliet Iwelunmor, Gbenga Ogedegbe, Lisa Dulli, Angela Aifah, Ucheoma Nwaozuru, Chisom Obiezu-Umeh, Deborah Onakomaiya, Ashlin Rakhra, Shivani Mishra, Calvin L. Colvin, Ebenezer Adeoti, Okikiolu Badejo, Kate Murray, Henry Uguru, Gabriel Shedul, Erinn M. Hade, Daniel Henry, Ayei Igbong, Daphne Lew, Geetha P. Bansal, Dike Ojji

**Affiliations:** 1 Department of Behavioral Science and Health Education, College for Public Health and Social Justice, Saint Louis University, St. Louis, USA; 2 Department of Population Health, New York University Grossman School of Medicine, New York, NY USA; 3 Institute for Excellence in Health Equity, New York University Grossman School of Medicine, New York, NY USA; 4 Family Health International 360, Durham, USA; 5 Department of Implementation Science, Wake Forest School of Medicine, Winston-Salem, NC USA; 6 Vilcek Institute of Graduate Biomedical Sciences, New York University Grossman School of Medicine, New York, NY USA; 7 Cardiovascular Research Unit, University of Abuja and University of Abuja Teaching Hospital, Gwagwalada, Abuja, Nigeria; 8 Department of Family Medicine, University of Abuja Teaching Hospital, Gwagwalada, Abuja, Nigeria; 9 Washington University School of Medicine, Washington University in St. Louis, St. Louis, USA; 10 Fogarty International Center, NIH, Bethesda, USA; 11 Department of Internal Medicine, Faculty of Clinical Sciences, College of Health Sciences, University of Abuja, Gwagwalada, Abuja, Nigeria

**Keywords:** Hypertension, Task-strengthening

## Abstract

**Background:**

Hypertension (HTN) is highly prevalent among people living with HIV (PLHIV), but there is limited access to standardized HTN management strategies in public primary healthcare facilities in Nigeria. The shortage of trained healthcare providers in Nigeria is an important contributor to the increased unmet need for HTN management among PLHIV. Evidence-based TAsk-Strengthening Strategies for HTN control (TASSH) have shown promise to address this gap in other resource-constrained settings. However, little is known regarding primary health care facilities’ capacity to implement this strategy. The objective of this study was to determine primary healthcare facilities’ readiness to implement TASSH among PLHIV in Nigeria.

**Methods:**

This study was conducted with purposively selected healthcare providers at fifty-nine primary healthcare facilities in Akwa-Ibom State, Nigeria. Healthcare facility readiness data were measured using the Organizational Readiness to Change Assessment (ORCA) tool. ORCA is based on the Promoting Action on Research Implementation in Health Services (PARIHS) framework that identifies evidence, context, and facilitation as the key factors for effective knowledge translation. Quantitative data were analyzed using descriptive statistics (including mean ORCA subscales). We focused on the ORCA context domain, and responses were scored on a 5-point Likert scale, with 1 corresponding to disagree strongly.

**Findings:**

Fifty-nine healthcare providers (mean age 45; standard deviation [SD]: 7.4, 88% female, 68% with technical training, 56% nurses, 56% with 1–5 years providing HIV care) participated in the study. Most healthcare providers provide care to 11–30 patients living with HIV per month in their health facility, with about 42% of providers reporting that they see between 1 and 10 patients with HTN each month. Overall, staff culture (mean 4.9 [0.4]), leadership support (mean 4.9 [0.4]), and measurement/evidence-assessment (mean 4.6 [0.5]) were the topped-scored ORCA subscales, while scores on facility resources (mean 3.6 [0.8]) were the lowest.

**Conclusion:**

Findings show organizational support for innovation and the health providers at the participating health facilities. However, a concerted effort is needed to promote training capabilities and resources to deliver services within these primary healthcare facilities. These results are invaluable in developing future strategies to improve the integration, adoption, and sustainability of TASSH in primary healthcare facilities in Nigeria.

**Trial registration:**

NCT05031819.

Contributions to the literature

- Although the need for integrated HIV/NCD care is recognized, evidence supporting context-specific strategies in sub-Saharan Africa (SSA) is limited.
- Task-strengthening strategies may mitigate important context barriers to optimal hypertension control in SSA. Shifting NCD risk assessment, diagnosis, and management from physicians to nurses is a viable and cost-effective strategy that can be adopted for HTN control in SSA and LMICs.
- Findings will inform a practice facilitation strategy to enhance the implementation and integration of evidence-based task-strengthening strategies for hypertension control among patients living with HIV in primary health facilities in Akwa-Ibom State, Nigeria.

## Introduction

Nigeria has a high burden of HIV and hypertension (HTN), with over a quarter of adults faced with a dual burden of hypertension and human immunodeficiency virus (HIV) [1]. The declining prevalence of infectious diseases and the increase in cardiovascular diseases has resulted in an epidemiological transition, with implications for strengthening health systems [1]. Most health systems in low-income countries like Nigeria are often ill-prepared, with limited resources or capacity to effectively coordinate chronic care efforts to benefit patients [2]. HIV programs have successfully established longitudinal care models, focusing on continuity and retention, routine monitoring, and healthy lifestyle promotion [3]. However, it remains unknown whether these successful outcomes can be translated to improve the quality and efficiency of care among hypertensive PLHIV [3]. In a literature review of models for integrated service delivery, major challenges with integration included human resources, the supply chain for non-communicable diseases management, poor referral systems, limited patient education, poor records keeping, and monitoring and evaluation [4, 5]. As numerous efforts are underway to address the growing HTN burden among PLHIV, one important precursor to the successful implementation of interventions is organizational readiness for change.

Defined as the extent to which organizations are willing (motivation) and able (capacity) to implement change [6], organizational readiness is associated with implementation success [7]. It plays an important role during all phases of implementation, reflecting an organization’s overall commitment, motivation, and capacity for change over time [6, 7]. Specifically, during the pre-implementation phase, readiness can guide the selection of implementation strategies to suit a particular context and needs of a population [8]. It can also highlight an organization’s commitment and collective capability during and post-implementation. It also identifies critical areas of the health system to enable informed scale-up of integrated HIV and non-communicable disease care services for PLHIV. According to prior research, examining readiness can help to explain why some efforts to implement interventions succeed, while others fail [9–11]. Organizations with greater resources are more likely to have quality implementation [9, 12]. However, poor or limited readiness may lead to resistance and reduce the effectiveness of the implementation process [10, 13].

In the context of integrating hypertension care within HIV service delivery, the use of guideline-concordant practices requires preparation, motivation, and investment of time and resources [14]. Identifying potential obstacles may enable the tailoring of the intervention based on available resources [15]. While evidence-based approaches, such as task-strengthening (whereby specific duties are transferred to health workers with shorter training or fewer qualifications), thereby increasing their effectiveness, are well known for HTN management and care [16, 17], implementation of such approaches has been sub-optimal. To our knowledge, organizational readiness has not been considered to integrate evidence-based HTN interventions within HIV care in Nigeria using evidence-based task-strengthening approaches. Therefore, to increase the likelihood of successful adoption and maintenance of evidence-based nurse-led Task-Strengthening Strategy for HTN control (TASSH) within primary healthcare centers in Akwa-Ibom, Nigeria, the current study sought to identify gaps and highlight factors and/or barriers associated with organizational readiness with the integration of an evidence-based task-strengthening strategy within HIV care in Nigeria.

## Methods

### Study setting and participants

We conducted a descriptive, cross-sectional study of primary health centers (PHCs) across Akwa-Ibom state in the southern region of Nigeria. This study is a self-standing part of a larger implementation trial aiming to investigate the effectiveness of practice facilitation (PF) in integrating evidence-based TASSH for the management of HTN at PHCs serving PLHIV. TASSH includes identifying HIV patients with uncontrolled HTN, initiating lifestyle counseling and medication treatment, and referring patients with complicated HTN for further care. Components of the PF strategy include training the practice facilitators using a train-the-trainer model to identify PHC site champions and coordinators to provide support in implementing TASSH based on the Nigerian national guidelines for the management of HTN in PHCs [18], building consensus for quality improvement targets, implementation of practice changes to support TASSH implementation, and Peer-to-Peer collaboration.

**Fig. 1:**
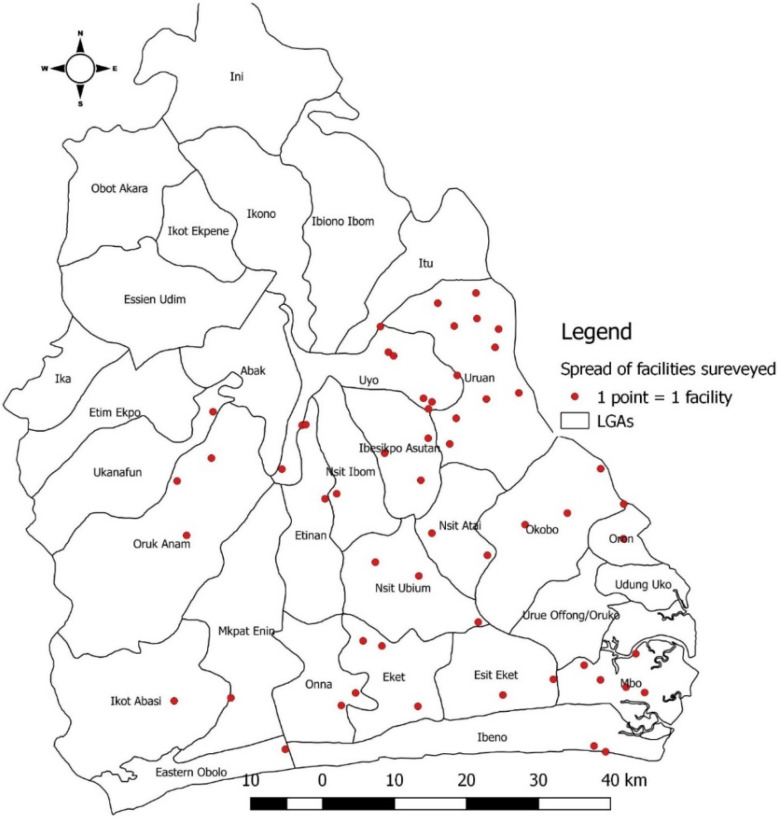
Spot map of locations of health facilities studied in Akwa Ibom State, Nigeria

TASSH has been successfully implemented across 32 community health centers in Ghana and led to 34% greater systolic blood pressure (BP) reduction than health insurance coverage alone [18, 19]. We purposively selected 59 primary healthcare facilities (Fig. 1) in Akwa-Ibom to reflect variations in the provision of comprehensive antiretroviral therapy (ART) services at the clinic sites, patient load, and facility type (private or public/government, or faith-based organizations), and primary facility level. Surveys were distributed to heads of health facilities and relevant outpatient departments such as outpatient, antenatal, family planning, child welfare, pharmacy, and laboratory. Survey distribution included efforts to reach the evening and night-shift employees and a representative sample of different types of providers. One response from each facility was provided. Ethical approval for this study was obtained from the University of Abuja Teaching Hospital Institutional Review Board. Written informed consent was obtained from the study participants.

### Data collection

The study survey was conducted using interviewer-administered questionnaires with a secure web-based data capture (Research Electronic Data Capture (REDCap) software) [20]. The survey instrument included a short series of questions to assess the facility respondent's sociodemographic characteristics, including age, gender, the highest level of education, professional designation, and the number of years of providing HIV care, as well as the health facility characteristics, including the number of HIV patients seen per month, cost of HTN care, and Organizational Readiness to Change Assessment (ORCA) tool [21] to assess healthcare facility readiness [1] quantitatively. ORCA is based on the Promoting Action on Research Implementation in Health Services (PARIHS) framework and measures evidence, context, and facilitation as the critical factors for effective knowledge translation [22, 23]. The ORCA assesses aspects of an organization’s willingness to adopt intervention or new practices and the capability to implement change [24]. The tool consists of three scales (evidence, context, and facilitation) with multiple subscales developed to assess elements of the PARIHS framework. ORCA was selected given that it is theory-grounded and has been used and validated as part of several evidence-based efforts in health facilities [21, 25]. For the study, we focused on the context scale to assess the organizational readiness of health facilities in Akwa-Ibom, Nigeria, to integrate an evidence-based task-strengthening strategy within their HIV care centers. The context scale measures the research or practice environment and consists of six subscales assessing organizational culture, leadership, and measurement [26, 27].

Organizational culture refers to the values, beliefs, and attitudes shared by members of the organization [21, 26, 28]. Leadership includes elements of teamwork, control, decision-making, effectiveness of organizational structures, and issues related to empowerment [21, 26, 28]. Measurement relates to how the organization measures its performance and how (or whether) feedback is provided to people within the organization, as well as the quality of measurement and feedback [21, 26, 28]. Specifically, two sub-scales assess organizational culture, two sub-scales assess leadership practice, and one sub-scale assesses measurement or evaluation in terms of setting goals, and tracking and communicating performance. In addition, context includes a sub-scale that measures resources to support practice changes [21]. Participant’s responses to these subscales were scored on a 5-point Likert scale “Always,” “Sometimes,” “Rarely,” “Never,” and “Do not Know.”

### Data analysis

Descriptive statistics were used to characterize all quantitative variables from the survey. Categorical variables were reported as frequencies and percentages. The ORCA responses were used to characterize health facility readiness. Mean and standard deviations of subscale scores were calculated among all study participants representing the health facilities. Scale and subscale scores are calculated by dividing the total score by the number of items on the scale, resulting in scores of 1 to 5. Higher scores demonstrate a more favorable attitude and reflect a greater readiness for change. Data management and statistical analysis were performed using the SAS System version 9.4 (SAS, Cary, NC, USA, 2016) [29].

## Results

### Study participants

Table 1 provides the sociodemographic characteristics of study participants. A total of 59 participants completed surveys; all healthcare professionals approached participated in our study with a mean age of 45 years (standard deviation [SD]: 7.4). The majority of the study participants were female, 52 (88%), and aged 35 years and older, 55 (93%). More than half of the participants, 33 (56%), were nurses, while the remaining consisted of physicians, 2 (3%), and other health professionals, 24 (41%). More than half of the participants, 33 (56%), had 1 to 5 years of experience providing HIV care.

**Table 1:** Sociodemographic characteristics of respondents (*N* = 59)

Characteristics	Frequency (*n*)	Percentages (%)
**Age (years)**		
26–30	1	1.7
31–35	3	5.1
35 +	55	93.2
**Gender**		
Male	7	11.9
Female	52	88.1
**Highest level of education**		
Technical training	40	67.8
Bachelors	17	28.8
Masters	2	3.4
**Professional designation**		
Physician	2	3.4
Nursing/midwifery	33	55.9
Others^a^	24	40.7
**Number of years of providing HIV care**		
1–5	33	55.9
6–10	24	40.7
11–15	2	3.4

Others^a^: Community Health Extension Worker (9), Senior Community Health Extension Worker (4), Chief Community Health Officer (1), Community Health Officer (1), Food Scientific Officer (1), Health Assistant (1), Health Record Officer (1), Medical Laboratory Technologist (1), Monitoring and Evaluation Officer (1), Nutrition Officer (1), Pharmacist (1), Pharmacy Technician (1), Principal Community Health Extension Worker (1)

### Primary healthcare facilities’ characteristics

Among the 59 primary healthcare facilities evaluated, nearly half (48%, *n* = 28) reported serving 11–30 HIV patients per month. Over half, 33 (56%) of the health facilities reported not seeing HIV patients with HTN, while 25 (42%) reported seeing one to ten HIV patients with HTN. More than a third of the health facilities, 22 (37%), reported always having anti-HTN drugs in their facilities, while 20 (33.9%) reported rarely having antihypertensive drugs available. The 1-month consultation fee for treating HTN in over a third of 23 (39%) of the health facilities was reported to be of no cost, while it was between 100 and 900 Naira (equivalent to $0.25–$2.00) in another third, 19 (32.2%), of the health facilities. Only 2 (3%) of the health facilities provided antihypertensive drugs at no cost to their patients from ongoing intervention programs. Table 2 provides additional information on the characteristics of the participating primary healthcare facilities.

**Table 2:** Current PHC characteristics with regard to HIV and HTN care (*N* = 59)

Variable	Frequency (*n*)	Percentages (%)
**Range of HIV patients seen in the facilities in a month**		
1–10	8	13.6
11–30	28	47.5
31–50	5	8.5
51–100	4	6.8
101–500	8	13.6
500 +	6	10.2
**Hypertensive HIV patients seen in facilities**		
Zero (none)	33	55.9
1–10	25	42.4
10 +	1	17
**Cost of treating hypertension in an HIV patient in 1 month in the facilities**		
No cost	23	39.0
100–900	19	32.2
1000–1900	10	16.9
2000–2900	3	5.1
3000–3900	3	5.1
4000 +	1	1.7

### Participating health facilities’ readiness to implement TASSH: ORCA context scale

**Fig. 2:**
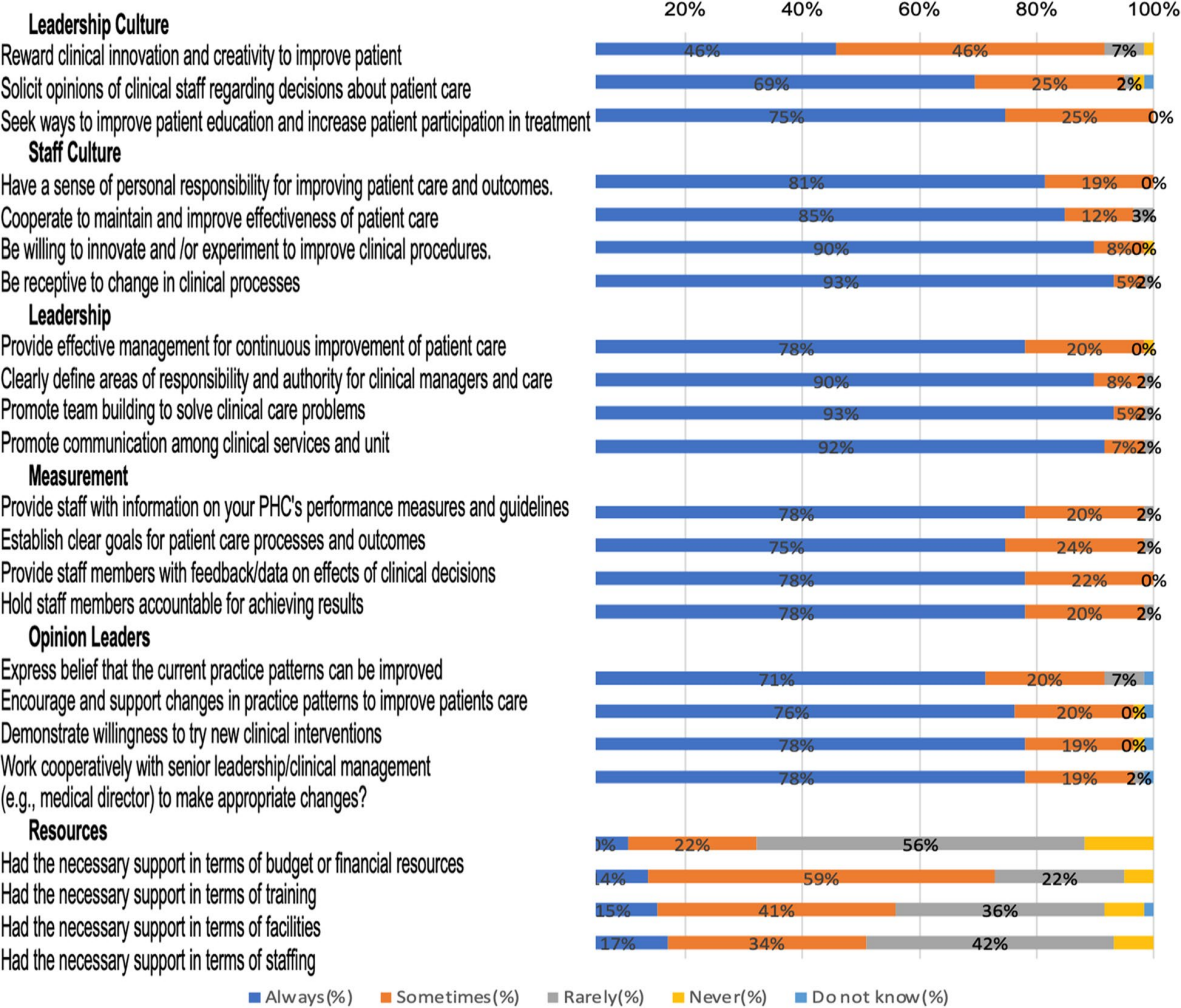
The proportion of responses for the context scale

The ORCA context scales are summarized in Table 3. For each of the six subscales that make up the context scales (leadership culture, staff culture, leadership, measurement, opinion leaders, and resources), the mean scores were generally high, with 5/6 of the sub-scales having an average of 4.00 or higher. Leadership (mean [SD]: 4.57 [0.41]), staff culture (4.85 [0.42]), measurement or evidence-assessment (4.76 [0.46]), opinion leaders (4.67 [0.73]), and leadership culture (4.57 [0.63]) were the top-scored context subscales, while resources scored the lowest with a mean score of (3.59 [0.82]). Proportions related to the context scale are provided in Fig. 2.

**Table 3:** Reporting of context scale (*n* = 59)

Sub-scale	Context assessment scale	Mean score	Standard deviation	Mode score
**Leadership Culture**	a. Reward clinical innovation and creativity to improve patient	4.36	0.69	4
	b. Solicit opinions of clinical staff regarding decisions about patient care	4.59	0.77	5
	c. Seek ways to improve patient education and increase patient participation in treatment	4.75	0.44	5
	**Subscale mean**	**4.57**	**0.63**	
**Staff Culture**	a. Have a sense of personal responsibility for improving patient care and outcomes	4.81	0.39	5
	b. Cooperate to maintain and improve the effectiveness of patient care	4.81	0.47	5
	c. Be willing to innovate and experiment to improve clinical procedures	4.86	0.47	5
	d. Be receptive to change in clinical processes	4.92	0.34	5
	**Subscale mean**	**4.85**	**0.42**	
**Leadership**	a. Provide effective management for continuous improvement of patient care	4.75	0.54	5
	b. Clearly define areas of responsibility and authority for clinical managers and care	4.88	0.38	5
	c. Promote team building to solve clinical care problems	4.92	0.34	5
	d. Promote communication among clinical services and unit	4.90	0.36	5
	**Subscale mean**	**4.86**	**0.41**	
**Measurement**	a. Provide staff with information on your PHC’s performance measures and guidelines	4.76	0.47	5
	b. Establish clear goals for patient care processes and outcomes	4.73	0.49	5
	c. Provide staff members with feedback/data on effects of clinical decisions	4.78	0.42	5
	d. Hold staff members accountable for achieving results	4.76	0.47	5
	**Subscale mean**	**4.76**	**0.46**	
**Opinion Leaders**	a. Express belief that the current practice patterns can be improved	4.59	0.77	5
	b. Encourage and support changes in practice patterns to improve patient care	4.68	0.73	5
	c. Demonstrate the willingness to try new clinical interventions	4.69	0.73	5
	d. Work cooperatively with senior leadership/clinical management (e.g. medical director) to make appropriate changes?	4.71	0.67	5
	**Subscale mean**	**4.67**	**0.73**	
**Resources**	a. Had the necessary support in terms of budget or financial resources	3.31	0.81	3
	b. Had the necessary support in terms of training	3.81	0.73	4
	c. Had the necessary support in terms of facilities	3.61	0.89	4
	d. Had the necessary support in terms of staffing	3.61	0.85	3
	**Subscale mean**	**3.59**	**0.82**

## Discussion

Our descriptive study utilized the ORCA tool to measure organizational readiness, particularly contextual factors for implementing TASSH in primary healthcare facilities in Akwa Ibom, Nigeria. This study adds to a growing body of research about organizational readiness for health practices and intervention integration into healthcare settings. Evaluating the context (culture and quality of the environment in which the intervention would be implemented) can improve the process and implementation outcomes [30] by identifying alignment areas and potential barriers. We encountered largely positive feedback on organizational readiness with several notable recommendations to facilitate PF in the integration of evidence-based TASSH for the management of HTN at PHCs serving PLHIV. Study participants perceived leadership, the organizational culture of staff members, and the measurement of evidence-based assessments as salient and conducive in their health facilities for the integration of TASSH. In contrast, resource scores had the lowest mean score and were less available for TASSH implementation [31].

Leadership and leadership culture indicators were largely positive and rated highly among study participants. Research has shown that leadership is an important contextual dimension [32, 33] and valuable in encouraging innovation and adoption of evidence-based interventions within organizations. Leadership support, motivation, and receptivity to innovations facilitate processes critical for implementation success [34, 35] by helping orchestrate decision processes from adoption to sustainability. The implementation strategy for TASSH integration within health facilities would involve working closely with health facility leaders to tailor approaches that best fit each context. Notably, although the overall mean score for leadership was relatively high, less than half of the participants agreed that their health facilities “always” reward clinical innovation and creativity for improvement. Further studies would be needed to elucidate preferences for rewards and how that is conceptualized by leadership and healthcare workers within organizations. Such evidence bolsters the demand for actively engaging key stakeholders to successfully integrate HTN/HIV services.

In addition, the influence of opinion leaders within the participating health facilities was scored highly. Opinion leaders are individuals who can influence the opinions, attitudes, beliefs, motivations, and behaviors of others [36]. Opinion leaders play a critical role in promoting the adoption of innovations and evidence-based interventions within organizations [37]. Opinion leaders, also known as “champions” in implementation science literature, play a crucial role in shaping organizational change by building organizational support for new practices and facilitating the growth of organizational coalitions in support of implementation [38–40]. To integrate TASSH within primary health facilities, the research team can tap into this opportunity by working with health facilities to identify opinion leaders or “champions” among the health providers. It is important to note that operating as an opinion leader to implement interventions within health facilities is a challenging and multifaceted role [40]. Therefore, adequate training and resources would be needed to ameliorate the additional burden associated with this tasking role.

Consistent with previous literature, the available resource subscale had the lowest ORCA mean score [31, 41]. In other studies in sub-Saharan Africa, lacking critical resources such as BP machines, medicines for HTN, and information on HTN/HIV integration hindered the successful integration of HTN care in HIV clinics [31, 42]. The lower scores for general resources suggest that many facilities may require additional staffing and financial resources to implement antihypertensive stewardship initiatives successfully. This underscores the need to adapt TASSH within the available resources while leveraging the strengths of the health facilities.

Our study collectively highlights several key contextual factors that can be leveraged to integrate practice facilitation and TASSH within health facilities in Nigeria. First, the study shows strong leadership support within the health facilities. Leadership buy-in is integral in the adoption and implementation of interventions. Second, we identified potential staff enthusiasm for the intervention implementation based on high scores on staff culture. This study provides a starting point for identifying favorable resources and opportunities for adopting TASSH and specific areas that may require targeted monitoring or assistance throughout the implementation cycle (pre-implementation and full-scale implementation) [29].

### Strengths

Our study provides a systematic approach to engaging health facilities to determine readiness for intervention implementation. We achieved this by using ORCA, a valuable tool for healthcare organizations with evidence-based intervention implementation, identification of potential barriers, facilities, and opportunities for implementation, adoption, and ultimately sustainability.

### Study limitations

However, our findings should be interpreted with caution, as the study design, including a sample of fifty-nine health facilities, limits generalizability to other primary health facilities in the State of Akwa Ibom, Nigeria. Nonetheless, we ensured that our sample was diverse regarding their occupation and involvement in the primary health facilities. Secondly, perspectives and practices are rapidly evolving across health facilities, given ever-changing inner and outer setting factors [43]. However, this study only focused on a one-time cross-sectional assessment. Thirdly, this study does not capture patient perspectives and all healthcare providers in the health facilities. In addition, there is a possibility for a potential responder bias about social desirability, perceived leadership, and resources given that the survey was interviewer-administered. It is possible that participants who were less engaged in the health facilities did not participate in the assessment, and, as a result, their perspectives were not captured.

## Conclusions

There is a growing evidence base on the benefits of integrating HTN care within HIV clinics. Evaluating organizational readiness is a critical prerequisite for the tailored implementation of evidence-based interventions and practices [41]. Our study uses the ORCA tool for a systematic evaluation to understand the common barriers, facilitators, and opportunities for implementing TASSH within primary healthcare facilities, to optimize the chance of successful adoption and integration. Overall, findings show organizational support for innovation and the health providers at the participating health facilities. However, a concerted effort is needed to promote training capabilities and resources to deliver services within these primary healthcare facilities. These results are invaluable in developing future strategies to improve the integration, adoption, and sustainability of TASSH in primary healthcare facilities in Nigeria. Future implementation strategies for TASSH in Nigeria should consider these factors and tailor interventions accordingly.

## Data Availability

Data is available upon request to the corresponding author.
